# Classifying invasive lobular carcinoma as special type breast cancer may be reducing its treatment success: A comparison of survival among invasive lobular carcinoma, invasive ductal carcinoma, and no-lobular special type breast cancer

**DOI:** 10.1371/journal.pone.0283445

**Published:** 2023-07-10

**Authors:** Rusen Cosar, Necdet Sut, Sernaz Topaloglu, Ebru Tastekin, Dilek Nurlu, Talar Ozler, Eylül Şenödeyici, Melisa Dedeli, Mert Chousein, Irfan Cicin

**Affiliations:** 1 Department of Radiation Oncology, Faculty of Medicine, Trakya University, Edirne, Turkey; 2 Department of Biostatistics, Faculty of Medicine, Trakya University, Edirne, Turkey; 3 Department of Medical Oncology, Faculty of Medicine, Trakya University, Edirne, Turkey; 4 Department of Pathology, Faculty of Medicine, Trakya University, Edirne, Turkey; 5 Faculty of Medicine, Trakya University, Edirne, Turkey; Kimura Hoospital, JAPAN

## Abstract

**Purpose:**

The literature contains different information about the prognosis of invasive lobular carcinoma of breast cancer (BC). We aimed to address the inconsistency by comparatively examining the clinical features and prognosis of invasive lobular carcinoma patients in our university and to report our experience by dividing our patients into various subgroups.

**Patients and methods:**

Records of patients with BC admitted to Trakya University School of Medicine Department of Oncology between July 1999 and December 2021 were reviewed. The patients were divided into three groups (No-Special Type BC, Invasive Lobular Special Type BC, No-Lobular Special Type BC). Patient characteristics, treatment methods and oncological results are presented. Survival curves were generated using the Kaplan–Meier method. Statistical significance of survival among the selected variables was compared by using the log-rank test.

**Results:**

The patients in our study consisted of 2142 female and 15 male BC patients. There were 1814 patients with No-Special Type BC, 193 patients with Invasive Lobular Special Type BC, and 150 patients with No-Lobular Special Type BC. The duration of disease-free survival (DFS) was 226.5 months for the No-Special Type BC group, 216.7 months for the No-Lobular Special Type BC group, and 197.2 months for the Invasive Lobular Special Type BC group, whereas the duration of overall survival (OS) was 233.2 months for the No-Special Type BC group, 227.9 for the No-Lobular Special Type BC group, and 209.8 for the Invasive Lobular Special Type BC group. The duration of both DFS and OS was the lowest in the Invasive Lobular Special Type BC group. Multivariate factors that were significant risk factors for OS were Invasive Lobular Special Type BC histopathology (p = .045), T stage, N stage, stage, skin infiltration, positive surgical margins, high histological grade, and mitotic index. Modified radical mastectomy, chemotherapy, radiotherapy and use of tamoxifen and aromatase inhibitors for more than 5 years were significant protective factors for overall survival.

**Conclusion:**

The histopathological subgroup with the worst prognosis in our study was Invasive Lobular Special Type BC. Duration of DFS and OS were significantly shorter in Invasive Lobular Special Type BC than No-Lobular Special Type BC group. The classification of Invasive Lobular BC under the title of Special Type BC should be reconsidered and a more accurate treatment and follow-up process may be required.

## Introduction

Invasive lobular breast cancer (ILC) is the most common special histological type of breast cancer (BC). While ILC accounts for 5% of invasive carcinomas, its incidence has increased up to 10–14% with the developments in diagnostic methods and novel discoveries. However, ILC remains less common in Asian populations (2–6%) [1–6]. As the incidence of ILC is significantly less than invasive ductal carcinoma (IDC), the most common histopathological subtype of BC, its clinical and prognostic features and biological behavior become clearer as more studies are published [7–15].

ILC is within the Special type of BC group, along with tubular, mucinous, papillary, micropapillary, medullary, metaplastic, and apocrine histopathological subtypes, whereas IDC is among the No-Special Type BC group composed of highly heterogeneous subtypes [10]. ILC stands out among the other histopathological subtypes in the Special type of BC group with its distinct clinical course, prognosis, and biological features. While the survival rate of ILC was better than or similar to that of IDC in series with less than 6 years of follow-up, the prognosis of ILC was found to be worse than IDC in series with longer follow-up. However, the St Gallen international expert consensus guidelines and the National comprehensive cancer network (NCCN) recommend that ILC should be treated with the same treatment paradigms as IDC, despite their many different features. Therefore, systemic treatment decisions for ILC and IDC are often similar [10]. Highlighting ILC as the subgroup with better prognosis in the series published in the past years may have prevented the treatment decision from being more aggressive, resulting in worse survival than IDC in the long term [6–9, 11–14].

Treating the “Special Type” ILC similarly to the “No-Special Type” IDC may cause the clinicians to overlook important details regarding this patient group [13]. However, larger tumor diameter, more lymph node metastases, high hormone receptor positivity, loss of E-cadherin and the potential of atypical metastasis are among the currently known distinct features of ILC [3, 4]. Unlike IDC, ILC shows different growth patterns and biological features, rather than masses that can easily be diagnosed with palpation or mammography. Additionally, an increased rate of multiple metastases, low rates of pathological complete response to neoadjuvant chemotherapy, and frequent positive surgical margins are among the features that make ILC more remarkable [14, 15].

Different information in the literature regarding prognosis has led us to comparatively examine the clinical features and prognosis of ILC patients in our series. We aimed to determine our own patient characteristics and report our treatment experience of ILC by dividing our patient series into various subgroups.

## Material and methods

### Patient characteristics

Patients with BC who applied to Trakya University School of Medicine Departments of Radiation Oncology and Medical Oncology between July 1999 and December 2021 were retrospectively analyzed. Approval was obtained from the Human Research Ethics Committee of Trakya University Medical Faculty Hospital (TUTF-BAEK 2022/170) for the use of patient information in the study. The consent form was submitted to the local ethics committee (Trakya University Faculty of Medicine Dean’s Non-Invasive Scientific Research Ethics Committee, Edirne, Turkey). Informed consent forms were prepared in accordance with the Declaration of Helsinki. In the study, permission was obtained from the patients, and if the patient died, from the legal guardians of the patients, by signing a written consent form, to use the information in the registry files containing the patient information.

Medical records and pathological reports were retrospectively converted into SPSS data to evaluate the clinicopathological features. After excluding 190 patients with ductal carcinoma in situ and lobular carcinoma in situ from a total of 2347 BC patients, the remaining 2157 patients with invasive carcinoma were included in the study. The patients in our series consisted of 2142 female and 15 male BC patients. No-Special Type BC (Completely invasive ductal carcinoma, IDC) and Special Type BC were divided as invasive lobular carcinoma (ILC), mixed type (IDC+ILC), epidermoid carcinoma, mucinous carcinoma, medullary carcinoma, papillary carcinoma, tubular carcinoma, adenoid cystic carcinoma, secretory carcinoma, apocrine carcinoma, and metaplastic carcinoma. Later, the patients were divided into three groups: No-Special Type BC (IDC) (n = 1814), Invasive Lobular Special Type BC (ILC, ILC+IDC mixed type) (n = 193), and No-Lobular Special Type BC (n = 150). Disease-free survival (DFS) and overall survival (OS) analyzes of the patient groups were performed. Patient characteristics were tabulated as ratios and numbers, and comparisons were made between groups. Finally, univariate, and multivariate analyzes of factors affecting DFS and OS were performed.

### Clinicopathological features

Pathological and clinical staging in our series was performed according to the seventh edition of the American Joint Committee on Cancer Staging Manual [16]. No-Special Type BC, Invasive Lobular Special Type BC, No-Lobular Special Type BC, and histopathological diagnoses were evaluated using hematoxylin-eosin staining by pathologists specializing in BC at Trakya University School of Medicine Department of Pathology. Estrogen receptor (ER) and progesterone receptor (PR) positivity were determined by immunohistochemical staining. Hormone receptor positivity was defined as an ER score greater than or equal to 3 on the Allred Score [17]. HER2 positive was defined as a Herceptest score of 3+ or a Herceptest score of 2+ followed by fluorescent in situ hybridization (FISH) positive [18, 19]. Luminal type was defined as ER positive and HER2 negative. Histological grading was done using the Nottingham histological grading system. Pathological staging for extensive intraductal carcinoma (EIC), lymphovascular invasion (LVI), and perineural invasion (PNI) was done according to the seventh edition of the American Joint Committee on Cancer Staging Manual [17].

### Statistical analysis

Numerical results are expressed as the mean ± standard deviation, and categorical results are shown as n (%). Kaplan-Meier method was used to generate the survival curves. Log-rank test was used to compare the statistical significance of survival among the selected variables. Hazard ratios were estimated using univariate Cox regression analysis. Multivariate Cox regression analysis with backward elimination method was used to estimate hazard ratios and identify independent prognostic factors. All p values are two-sided, and *p<0*.*05* indicates statistical significance. Data analysis was performed using SPSS version 20.0 (IBM SPSS Statistics for Windows, Version 20.0. Armonk, NY: IBM Corp.).

## Results

**Fig 1:**
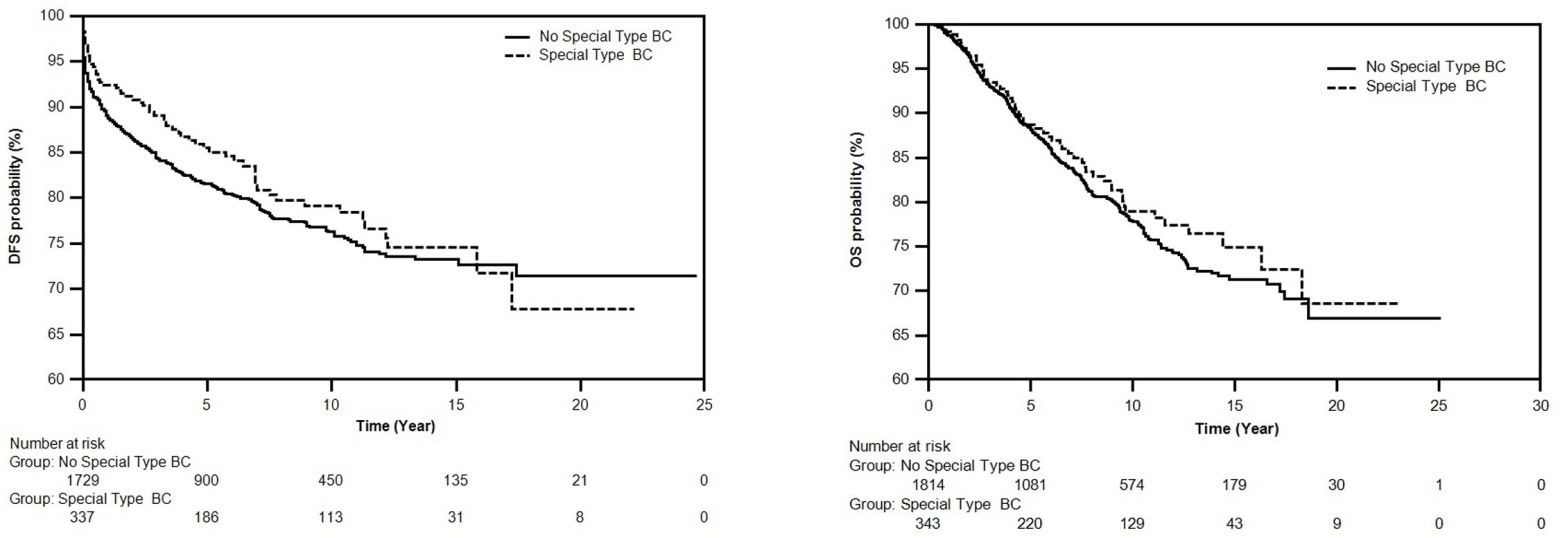
**A.** Survival curve DFS of No-Special Type BC and Special Type BC subgroups using Kaplan-Meier method. **B.** Survival curve of OS times of No-Special Type BC and Special Type BC subgroups using Kaplan-Meier method.

Out of 2157 patients, 1814 patients had No-Special Type BC and 342 patients had Special Type BC. No statistically significant difference between DFS and OS was found (Table 1 and Fig 1A and 1B).

**Table 1:** DFS and OS times, comparative log-rank test, p-value values of no-special type BC and special type BC subgroups obtained using Kaplan-Meier method.

		No-Special Type BC	Special Type BC	*P* value
				(Long-rank test)
**DFS**	Mean± SD	226,5 ± 3,4	208,2 ± 6,6	.234
	95% CI	219,8–233,1	195,2–221,2	
**OS**	Mean± SD	233,2 ± 3,5	221,8 ± 6,4	.379
	95% CI	226,3–240,1	209,2–234,4	

DFS: Disease free survival, OS: Overall survival, SD: Standard deviation, CI: Confidence Interval

**Fig 2:**
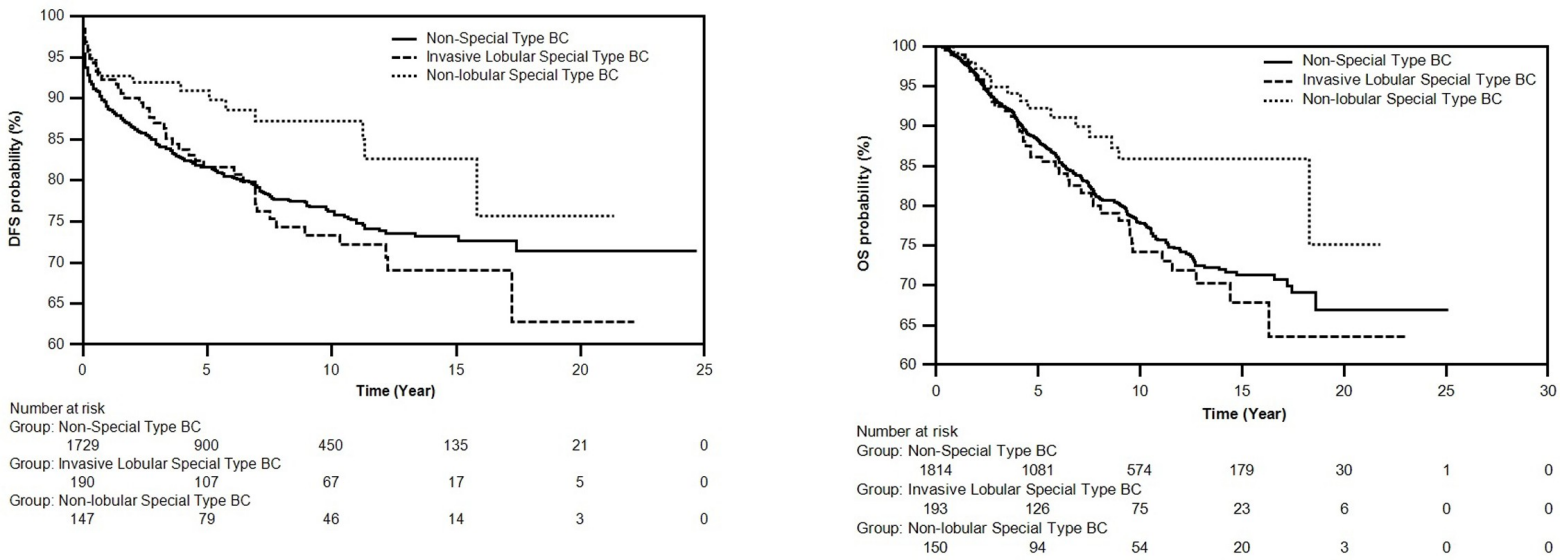
**A.** Survival curve of DFS times of No-Special Type BC and No-Lobular Special Type BC and Invasive Lobular Special Type BC subgroups using Kaplan-Meier method. There are 2066 patients at the beginning of the DFS curve, as 91 of a total of 2157 breast cancer patients had metastatic presentation at baseline. **B.** Survival curve of OS times of No-Special Type BC and No-Lobular Special Type BC and Invasive Lobular Special Type BC subgroups using Kaplan-Meier method.

In the second step, we divided our patient series into three groups as No-Special Type BC (n = 1814), Invasive Lobular Special Type BC (n = 193), No-Lobular Special Type BC (n = 150). The rate of ILC patients in our series was 8.9%. 4.1% (n = 8) of ILCs contained pleomorphic components. When all three groups were compared, the differences between the durations of both DFS and OS were statistically significant. The duration of DFS was 226.5 months for the No-Special Type BC group, 216.7 months for the No-Lobular Special Type group, and 197.2 months for the Invasive Lobular Special Type BC group, whereas the duration of OS was 233.2 months for the No-Special Type BC group, 227.9 months for the No-Lobular Special Type BC group, and 209.8 months for the Invasive Lobular Special Type BC group. The duration of both DFS and OS was the lowest in the Invasive Lobular Special Type BC group (Table 2 and Fig 2A and 2B).

**Table 2:** DFS and OS times, comparative log-rank test, p-value values of no-special type BC and no-Lobular Special Type BC, ILC subgroups obtained using Kaplan-Meier method.

DFS		No-Special Type BC (IDC)	No-Lobular Special Type BC	Lobular Special Type BC (ILC)
***P* value**	Mean±SD	226,5 ± 3,4	216,7±8,7	197,2 ± 8,9
**(Long-rank test)**	95% CI	219,8–233,1	199,6–233,7	179,6–214,7
**No-Special Type BC (IDC)** **Lobular Special Type BC** **(ILC)**			**.020**	
		
		.717	**.029**	
**OS**	Mean±SD	233,2 ± 3,5	227,9 ± 7,9	209,8±8,7
	95% CI	226,3–240,1	212,3–243,5	192,6–227,0
**No-Special Type BC (IDC)**			**.024**	.504
**Lobular Special Type BC (ILC)**			**.018**	

DFS: Disease free survival, OS: Overall survival, SD: Standard deviation, CI: Confidence Interval

**Fig 3:**
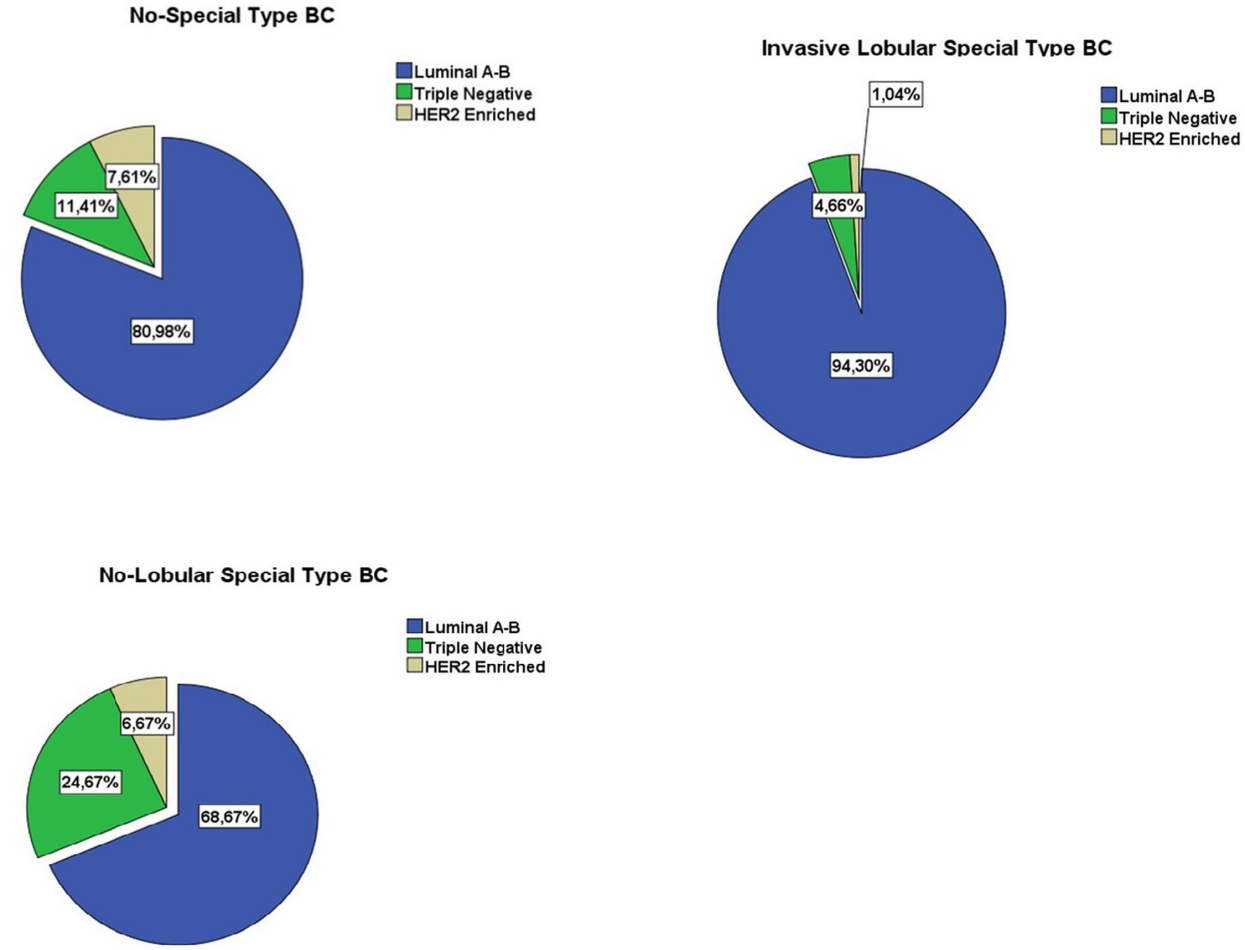
Subgroup distribution according to histopathological subgroup differentiation. **A**. No-Special Type BC, **B**. Invasive Lobular Special Type BC, **C**. No-Lobular Special Type BC.

The histopathological subgroup distribution, which consisted of ER, PR, Ki67 and CerbB2, was 80.98% Luminal AB, 11.41% Triple Negative, 7.1% HER2 Enriched for IDC, while No-Lobular Special Type was divided as 68.67% Luminal AB, 24.67% Triple Negative, 6.67% HER2 Enriched, and ILC was divided as 94.30% Luminal AB, 4.66% Triple Negative, 1.04% HER2 Enriched. The subgroup dominating the histopathological subgroup of ILC was Luminal A-B with a rate of 94.30% (Fig 3A–3C).

Patient characteristics of all three groups (No-Special Type BC, Invasive Lobular Special Type BC, No-Lobular Special Type BC) were analyzed (Table 3). Gender, age, menstruation, family history, histological type, tumor quadrant, T stage, N stage, stage, metastasis site, breast surgery type (modified radical mastectomy (MRM) / breast conserving surgery (BCS)), axillary surgery type (axillary curettage (AC) /sentinel lymph node dissection (SLND)), skin infiltration, surgical margin (in patients with positive surgical resection margin, re-excision was performed first and the surgical resection margin was positive despite this), tumors grade, mitotic index (MI), ER, PR, Ki67 (<15, ≥15), HER2, EIC, LVI, PNI, subgroup (Luminal A, Luminal B, Triple Negative, HER2 Enriched), presence of anti-hormonal therapy, duration of Tamoxifen (TAM) and Aromatase Inhibitor (AI) use, use of Herceptin, and type of chemotherapy (CT) [No CT, AC+TXT (doxorubicin and cyclophosphamide followed by paclitaxel), FAC (fluorouracil, doxorubicin, cyclophosphamide)-FEC (fluorouracil, epirubisin, cyclophosphamide), -TAC (docetaxel, doxorubicin, cyclophosphamide), FAC-FEC+TXT, Ribociclib + Palbociclib, CMF (Cyclophosphamide Methotrexate Fluorouracil)] were analyzed as numbers and rates. Fisher exact test was used to compare the patient characteristics of the three groups. In the same table was made for the statistically significant patient characteristics of the three groups (Table 3).

**Table 3:** Distribution of patient characteristics.

	No-Special Type BC (IDC)		Invasive Lobular Special Type BC (ILC)		No-lobular Special Type BC	
	N	%	N	%	n	%
**Gender**						
**Female**	1800	99.2	193	100	150	99.33
**Male**	15	0.8	0	0	0	0.7
**Age**	92	5.10%	11	5.7	9	6
**<35**						
**35–50**	696	38.4	85	44	58	38.7
**>50**	1026	56.6	97	50.3	83	55.3
**Menstruation State**						
**Premenopausal**	701	38.9	81	42	54	36.2
**Postmenopausal**	1099	61.1	112	58	95	63.8
**Presence of Family History**	536	29.5	54	28	37	24.7
**Histological Type**						
**IDC**	1761	97.1				
**ILC**			138	71.5		
**IDC+ILC**			55	28.5		
**Mucinous**					44	29.3
**Medullary**					34	22.7
**Papillary**					25	16.7
**Tubular**					12	8
**Adenoid Cystic**					4	2.7
**Secretory**					2	1.3
**Apocrine**					15	10
**Metaplastic**					11	7.3
**Epidermoid**					3	2
**Breast**						
**Right**	869	47.9	103	53.4	69	46
**Left**	885	48.8	82	42.5	75	50
**Bilateral**	60	3.3	8	4.1	6	4
Tumor Quadrant***						
**Inner**	351	19.3	44	22.8	40	26.7
**Outer**	1085	59.8	104	53.9	89	59.3
**Periareolar**	239	13.2	29	15	11	7.3
**Multifocal**	139	7.7	16	8.3	10	6.7
Tm Size*						
**T1**	609	33.6	54	28	51	34
**T2**	948	52.3	98	50.8	80	53.3
**T3**	135	7.4	26	**13.5**	15	10
**T4**	122	6.7	15	7.8	4	2.7
Number of Infiltrated Axillary Nodes**, ***						
**0**	736	40.6	74	38.3	90	60
**1–3**	476	26.2	59	30.6	29	19.3
**4–9**	394	21.7	32	16.6	21	14
**≥10**	208	11.5	28	14.5	10	6.7
Stage*, ***						
**I**	366	20.2	38	19.7	41	27.3
**II**	778	42.9	78	40.4	75	50
**III**	538	29.7	66	34.2	29	19.3
**IV**	132	7.3	11	5.7	5	3.3
**Site of Metastasis**						
**None**	1460	80.5	152	78.8	134	89.3
**Bone**	123	6.8	18	9.3	5	3.3
**Lung**	16	0.9	3	1.6	4	2.7
**Liver**	13	0.7	1	0.5	0	0
**Brain**	21	1.2	2	1	1	0.7
**Multiple**	181	10	16	8.3	6	4
Breast Surgery Type**, ***						
**MRM**	834	47.9	98	51.6	50	33.8
**BCS**	907	52.1	92	48.4	98	66.2
**Axillary Surgery Type**						
**SLND**	435	25	48	25.3	45	30.4
**AC**	1306	75	142	74.7	103	69.6
**Skin Infiltration**	147	8.1	14	7.3	7	4.7
**Positive Surgical Margin**	337	18.6	41	21.2	27	18
Histological Grade*, **, ***						
**1**	219	12.1	25	13	45	30
**2**	859	47.4	111	57.5	55	36.7
**3**	736	40.6	57	29.5	50	33.3
Mitotic index *, ***						
**1**	285	15.7	41	21.2	37	24.7
**2**	895	49.3	99	51.3	79	52.7
**3**	634	35	53	27.5	34	22.7
ER Positive*, **, ***	1434	79.1	177	91.7	101	67.3
PR Positive*, **, ***	1175	64.8	165	85.5	81	54
Ki67*, ***						
**<15**	603	33.3	86	44.6	65	43.3
**≥15**	1209	66.7	107	55.4	85	56.7
**HER2 Positive**	459	25.3	24	12.4	25	16.7
EIC Positive***	309	17	33	17.1	16	10.7
LVI Positive **, ***	881	48.6	99	51.3	45	30
PNI Positive *, **, ***	342	18.9	47	24.4	11	7.3
Subgroup*, **, ***						
**HER2 Enriched**	138	7.6	2	1	10	6.7
**Triple Negative**	207	11.4	9	4.7	37	24.7
**Luminal A**	483	26.6	79	40.9	50	33.3
**Luminal B**	986	54.4	103	53.4	53	35.3
Anti-Hormonal Treatment Received*, **, ***	1467	80.9	181	93.8	103	68.7
**Duration of TAM Use**						
N/A*, **, ***	1051	57.9	97	50.3	101	67.3
**≤ 5 years**	679	37.4	82	42.5	43	28.7
**>5 years**	84	4.6	14	7.3	6	4
**Duration of AI Use**						
N/A*, **	734	40.5	59	30.6	72	48
**≤ 5 years**	850	46.9	113	58.5	62	41.3
**>5 years**	230	12.7	21	10.9	16	10.7
Herceptin *Eligibility	380	20.9	20	10.4	23	15.3
**RT Received**	1589	87.6	167	86.5	129	86
**RT Type**						
N/A**, ***	215	11.9	26	13.5	20	13.3
**Breast Only**	515	28.4	43	22.3	69	46
**Locoregional**	1084	59.8	124	64.2	61	40.7
**CT Received**						
N/A**, ***	303	16.7	42	21.8	43	28.7
**Neoadjuvant**	236	13	20	10.4	10	6.7
**Adjuvant**	1275	70,3	131	67.8	97	64.7
CT Type*, **, ***						
**N/A**	303	16.7	42	21.8	43	28.7
**AC+TXT**	875	48.2	74	38.3	67	44.7
**FAC-FEC-TAC**	358	19.7	57	29.5	28	18
**FAC, FEC+TXT**	209	11.5	16	8.3	8	5.3
**Ribociclib+Palbociclib**	6	0.3	0	0	0	0
**Pertuzumab**	22	1.2	0	0	0	0
**CMF**	41	2.3	4	2.1	5	3.3
Recurrence**, ***	372	20.5	45	23.3	19	12.7
Death**, ***	328	18.1	43	22.3	16	10.7

ER: Estrogen Receptor, PR: Progesterone Receptor, HER2: Human Epidermal Growth Factor Receptor 2, EIC: Extensive Intraductal Carcinoma, LVI: Lymphovascular Invasion, PNI: Perineural Invasion, TMX: Tamoxifen, AI: Aromatase Inhibitor, RT: Radiotherapy, CT: Chemotherapy, AC: Axillary Curettage SLND: Sentinel Lymph Node Dissection, AC: Adriamycin, Cyclophosphamide, TXT: Taxotere, FAC: Cyclophosphamid, Adriamycin, 5-Fulourouracil, FEC: 5-Fulouracil, Epirubicine, Cyclophosphamide, TAC: Taxotere, Adriamycin, Cyclophosphamid, RIBO+PABLO: Ribociclib+ Palbociclib, CMF: Cyclophosphamide, Methotrexate, Fluorouracil.

* Pairwise comparison of all three groups with Fisher’s exact test (p < .0.5) in terms of patient characteristics (IDC/ILC).

** Pairwise comparison of all three groups with Fisher’s exact test (p < .0.5) in terms of patient characteristics (ILC/No-Lobular Special Type).

*** Pairwise comparison of all three groups with Fisher’s exact test (p < .0.5) in terms of patient characteristics (IDC/No-Lobular Special Type).

The distribution of patient characteristics of the 3 histopathological subgroups are shown in Table 3. Of the 193 patients in the Invasive Lobular Special Type BC group in our series, 138 were pure ILC, and 55 were IDC+ILC BC patients. All patients in the Invasive Lobular Special Type BC group were women, mostly over 50 years of age and in the postmenopausal period. There was no significant difference in the presence of family history and bilateral arrangement among the 3 groups. While the rate of MRM in breast surgery was 45.5% (n = 982), only 7 of the patients had skin-sparing mastectomy. Periareolar and multifocal localizations were slightly more common in the Invasive Lobular Special Type BC group. A pairwise comparison of patient characteristics of all three groups were done.

### No-Special Type BC / Invasive Lobular Special Type BC patient characteristics

T stage, PNI positivity, ER and PR positivity rates were higher in Invasive Lobular Special Type BC, while Ki67, CerbB2 positivity, MI (mitotic index) and histological grade rates were higher in No-Special Type BC. Subgroup, rate of anti-hormonal treatment use, duration of TAM and AI use, CT type, and metastasis location were the features that showed significant difference between Invasive Lobular Special Type BC and No-Special Type BC. Breast and axillary surgery type and surgical margin positivity were not different between the two histopathological groups (Table 3). Although not statistically significant, MRM was preferred in 47.9% of the No-Special Type BC and 51.6% of the Invasive Lobular Special Type BC patients. While 83.2% of BC patients with No-Special Type BC histopathology received chemotherapy, this rate was 78.2% in the Invasive Lobular Special Type BC histopathological subgroup, and this difference was calculated differently at the level of statistical significance.

### Invasive Lobular Special Type BC / No-Lobular Special Type BC patient characteristics

N stage, stage, surgery type (breast and axillary surgery type), PNI, LVI, subgroup, ER and PR positivity, HG, duration of anti-hormonal therapy, duration of TAM and AI use, RT type, CT type, recurrence/metastasis rates and mortality rates were significantly different in the Invasive Lobular Special Type BC group (Table 3). While the MRM rate was 51.6% more preferred in Invasive Lobular Special Type BC in surgical treatment, BCS was preferred with 66.2% in No-Lobular Special Type BC, and this difference was statistically significant. In No-Lobular Special Type BC, the rate of administration of chemotherapy was 71.3%, the histopathological subgroup in which the least chemotherapy was preferred.

### No-Special Type BC / No-Lobular Special Type BC patient characteristics

The quadrant where the tumor is located, T stage, N stage, stage, surgical type (breast and axillar surgery), PNI, LVI, EIC, subgroup, ER and PR positivity, Ki67 rate, CerbB2 positivity, HG, presence of anti-hormonal therapy, RT type, CT type, metastasis location, recurrence/metastasis rate and death rate were found to be significantly higher in the No-Special Type BC group. While MRM was preferred in 47.9% of patients with IDC histopathology, it was performed in 33.8% of No-Lobular Special Type BC patients. SLND was preferred in 25% of No-Special Type BC patients and 30.4% of No-Lobular Special Type BC patients, while axillary curettage was the preferred treatment option in 75% of No-Special Type BC patients and 69.6% of No-Lobular Special Type BC patients (Table 3).

Cox regression test was used to examine the histopathological subgroups and patient characteristics with these three different clinical and pathological features. Multivariate factors that were significant risk factors for DFS in our study were age, being in the postmenopausal period, multicentric location, T stage, stage, HER2 positivity and MI, while duration of TAM and AI use, and use of Herceptin were the significant protective factors for DFS (Tables 4 and 5).

**Table 4:** Univariate analysis results affecting DFS.

		Recurrence		Univariate Cox Regression	
		N/A	Present	p	HR (95% CI)
**Age**	**<35**	83 (4.8)	29(6.7)	1	(Reference)
	**36–50**	691 (40.2)	148 (33.9)	**.035**	**.652(.438-.970)**
	**>50**	947 (55)	259 (59.4)	.557	.891(.607–1.308)
**Gender**	**Female**	1714 (99.6)	428 (98.2)	**.001**	**3.265(1.622–6.574)**
	**Male**	7 (0.4)	8 (1.8)		
**Menstruation Status**	**Premenopausal**	688 (40.1)	148 (34.6)	**.006**	**1.320(1.081–1.611)**
	**Postmenopausal**	1026 (59.9)	280 (65.4)		
**Family History**	**Present**	513 (29.8)	114 (26.1)	.117	1.187(.958–1.469)
	**N/A**	1208 (70.2)	322 (73.9)		
**Histological Subtype Group– 1**	**No-Special Type BC**	1442 (83.8)	372 (85.3)	.237	.852(.653–1.1111)
	**Special Type B**	279 (16.2)	64 (14.7)		
**Histological Subtype Group– 2**	**No-Special Type BC**	1442 (83.8)	372 (20.5)	1	(Reference)
	**Invasive Lobular Special Type BC**	148 (8.6)	45 (10.3)	.724	1.057(.776–1.441)
	**No-lobular Special Type BC**	131 (7.6)	19 (4.4)	**.022**	**.584(.368-.925)**
**Arrangement**	**Unilateral**	1674 (97.3)	409 (93.8)	**.003**	**1.808(1.225–2.670)**
	**Bilateral**	47 (2.7)	27 (6.2)		
**Breast**	**Left**	841 (48.9)	201 (46.1)	1	(Reference)
	**Right**	833 (48.4)	208 (47.7)	.833	1.021(.841–1.240)
	**Bilateral**	47 (2.7)	27 (6.2)	**.003**	**1.828(1.223–2.732)**
	**Inner**	351 (20.4)	84 (19.3)	1	(Reference)
	**Outer**	1034 (60.1)	244 (56)	.954	1.007(.786–1.291)
**Tumor Quadrant**	**Periareolar**	222 (12.9)	57 (13.1)	.597	1.095(.782–1.533)
	**Multicentric**	114 (6.6)	51 (11.7)	**< .001**	**1.876(1.324–2.657)**
**T Stage**	**T1**	648 (37.7)	66 (15.1)	1	(Reference)
	**T2**	889 (51.7)	237 (54.4)	**< .001**	**2.401(1.827–3.154)**
	**T3**	131 (7.6)	45 (10.3)	**< .001**	**2.803(1.919–4.095)**
	**T4**	53 (3.1)	88 (20.2)	**< .001**	**12.650(9.156–17.479)**
**N Stage**	**N0**	818 (47.5)	82 (18.8)	1	(Reference)
	**N1**	478 (27.8)	86 (19.7)	**.001**	**1.658(1.225–2.244)**
	**N2**	293 (17)	154 (35.3)	**.000**	**4.808(3.676–6.289)**
	**N3**	132 (7.7)	114 (26.1)	**.000**	**6.817(5.130–9.060)**
**Stage**	**I**	422 (24.5)	23 (5.3)	1	(Reference)
	**II**	825 (47.9)	106 (24.3)	**.001**	**2.181(1.390–3.424)**
	**III**	469 (27.3)	164 (37.6)	**< .001**	**5.605(3.622–8.672)**
	**IV**	5 (0.3)	143 (32.8)	**< .001**	**104.241(66.093–164.408)**
**Site of Metastasis**	**N/A**	1719 (99.9)	27 (6.2)	1	(Reference)
	**Bone**	1 (0.1)	145 (33.3)	**< .001**	**167.63(110.55–254.18)**
	**Lung**	0 (0)	23 (5.3)	**< .001**	**159.811(90.86–281.08)**
	**Liver**	0 (0)	14 (3.2)	**< .001**	**174.24(90.66–334.87)**
	**Brain**	0 (0)	24 (5.5)	**< .001**	**179.70(102.60–314.73)**
	**Multiple**	1 (0.1)	203 (46.5)	**< .001**	**171.71(113.89–258.88)**
**ER**	**Positive**	1393 (80.9)	319 (73.2)	**< .001**	**1.476(1.194–1.824)**
	**Negative**	328 (19.1)	117 (26.8)		
**PR**	**Positive**	1167 (67.8)	254 (58.3)	**< .001**	**1.499(1.239–1.814)**
	**Negative**	554 (32.2)	182 (41.7)		
**HER2**	**Positive**	383 (22.3)	125 (28.7)	**< .001**	**.668(.542-.823)**
**EIC**	**N/A** **Present**	1459 (84.8) 262 (15.2)	340 (78) 96 (22)	**< .001**	**1.536(1.224–1.927)**
**LVI**	**Present**	809 (47)	216 (49.5)	.312	.908(.752–1.095)
	**N/A**	912 (53)	220 (50.5)		
**PNI**	**Present**	311 (18.1)	89 (20.4)	.580	.936(.742–1.182)
	**N/A**	1410 (81.9)	347 (79.6)		
**Ki67**	**<15**	670 (39)	84 (19.3)	**< .001**	**2.481(1.955–3.148)**
	**≥15**	1049 (61)	352 (80.7)		
**Mitotic Index**	**1**	342 (19.9)	21 (4.8)	1	(Reference)
	**2**	913 (53.1)	160 (36.7)	**< .001**	**2.739(1.738–4.318)**
	**3**	466 (27.1)	255 (58.5)	**< .001**	**8.114(5.197–12.669)**
**Histologic Grade**	**Grade I**	261 (15.2)	28 (6.4)	1	(Reference)
	**Grade II**	845 (49.1)	180 (41.3)	**.002**	**1.877(1.261–2.795)**
	**Grade III**	615 (35.7)	228 (52.3)	**< .001**	**3.223(2.176–4.774)**
**Skin Infiltration**	**Present**	82 (4.8)	86 (19.7)	**< .001**	**.214(.168-.272)**
	**N/A**	1639 (95.2)	350 (80.3)		
**Surgical Margin**	**Negative**	1387 (80.6)	365 (83.7)	.664	.945(.732–1.219)
	**Positive**	334 (19.4)	71 (16.3)		
**Subgroup (Luminal)**	**Luminal A-B**	1422 (82.6)	332 (76.1)	**1**	**(Reference)**
	**Triple Negative**	199 (11.6)	54 (12.4)	**.406**	**1.130(.847–1.506)**
	**HER2 Enriched**	100 (5.8)	50 (11.5)	**< .001**	**2.139(1.588–2.881)**
**Subgroup**	**HER2 Enriched**	100 (5.8)	50 (11.5)	1	(Reference)
	**Triple Negative**	199 (11.6)	54 (12.4)	**.001**	**.527(.359-.775)**
	**Luminal A**	547 (31.8)	65 (14.9)	**< .001**	**.246(.170-.356)**
	**Luminal B**	875 (50.8)	267 (61.2)	**.001**	**.597(.441-.808)**
**Surgery**	**N/A**	8 (0.5)	70 (16.1)	**< .001**	**.054(.041-.071)**
	**Present**	1713 (99.5)	366 (83.9)		
**Breast Surgery Type**	**BCS**	970 (56.6)	127 (34.7)	**< .001**	**2.049(1.652–2.542)**
	**MRM**	743 (43.4)	239 (65.3)		
**Axillary Surgery**	**N/A**	8 (0.5)	70 (16.1)	**< .001**	**.054(.041-.071)**
	**Present**	1713 (99.5)	366 (83.9)		
**Axillary Surgery Type**	**SLND**	481 (28.1)	47 (12.8)	**< .001**	**1.966(1.445–2.674)**
	**AK**	1232 (71.9)	319 (87.2)		
**Anti-Hormonal Treatment**	**Present**	1419 (82.5)	332 (76.1)	**.001**	**1.454(1.167–1.813)**
	**N/A**	302 (17.5)	104 (23.9)		
**Duration of TAM Use**	**N/A**	1059 (61.5)	190 (43.6)	1	(Reference)
	**≤ 5 years**	570 (33.1)	234 (53.7)	.000	1.885(1.555–2.284)
	**>5 years**	92 (5.3)	12 (2.8)	.071	.584(.326–1.047)
**Duration of AI Use**	**N/A**	669 (38.9)	196 (45)	1	(Reference)
	**≤ 5 years**	819 (47.6)	206 (47.2)	.075	.837(.688–1.018)
	**>5 years**	233 (13.5)	34 (7.8)	**< .001**	**.429(.298-.619)**
**RT**	**Present**	1579 (91.7)	306 (70.2)	**< .001**	3.580(2.914–4.398)
	**N/A**	142 (8.3)	130 (29.8)		
**RT Type**	**N/A**	134 (7.8)	127 (29.1)	1	(Reference)
	**Breast Only**	580 (33.7)	47 (10.8)	< .001	.124(.089-.174)
	**Locoregional**	1007 (58.5)	262 (60.1)	< .001	.350 (.283-.433)
**Herceptin Eligibility**	**Eligible**	331 (19.2)	92 (21.1)	**.023**	**.765(.607-.965)**
	**Ineligible**	1390 (80.8)	344 (78.9)		
**CT**	**N/A**	318 (18.5)	46 (10.6)	1	(Reference)
	**Neoadjuvant**	184 (10.7)	82 (18.8)	< .001	2.812(1.959–4.035)
	**Adjuvant**	1219 (70.8)	308 (70.6)	.025	1.424(1.044–1.943)
**CT** **Type**	**N/A**	328 (19.1)	60 (13.8)	1	(Reference)
	**AC+TXT**	863 (50.1)	153 (35.1)	.986	.997(.740–1.344)
	**FAC-FEC-TAC**	310 (18)	132 (30.3)	.015	1.465(1.077–1.994)
	**FAC-FEC+TXT**	**189 (11)**	**44 (10.1)**	.932	.983(.666–1.452)
	**RIBO+PABLO**	**0 (0)**	**6 (1.4)**	< .001	20.437(8.755–47.704)
	**PERJETA**	**2 (0.1)**	**20 (4.6)**	< .001	16.309(9.751–27.276)
	**CMF**	**29 (1.7)**	**21 (4.8)**	< .001	2.663(1.619–4.379)

ER: Estrogen Receptor, PR: Progesterone Receptor, HER2: Human Epidermal Growth Factor Receptor 2, EIC: Extensive Intraductal Carcinoma, LVI: Lymphovascular Invasion, PNI: Perineural Invasion, TMX: Tamoxifen, AI: Aromatase Inhibitor, RT: Radiotherapy, CT: Chemotherapy, AC: Axillary Curettage, SLND: Sentinel Lymph Node Dissection, AC: Adriamycin, Cyclophosphamide, TXT: Taxotere, FAC: Cyclophosphamid, Adriamycin, 5-Fulourouracil, FEC: 5-Fulouracil, Epirubicine, Cyclophosphamide, TAC: Taxotere, Adriamycin, Cyclophosphamid, RIBO+PABLO: Ribociclib+ Palbociclib, CMF: Cyclophosphamide, Methotrexate, Fluorouracil

**Table 5:** Multivariate analysis results affecting DFS.

		Recurrence		Multivariate Cox Regression	
		N/A	Present	p	HR (95% CI)
**Age**	**<35**	83 (4.8)	29 (6.7)	1	(Reference)
	**36–50**	691 (40.2)	148 (33.9)	**.05**	**.629(.395–1.000)**
	**>50**	947 (55)	259 (59.4)	.175	.671(.377–1.194)
**Menstruation Status**	**Premenopausal**	688 (40.1)	148 (34.6)	.**025**	1.524(1.054–2.205)
	**Postmenopausal**	1026 (59.9)	280 (65.4)		
**Histological Subtype Group- 2**	**No-Special Type BC**	1442 (83.8)	372 (85.3)	1	(Reference)
	**Invasive Lobular Special Type BC**	148 (8.6)	45 (10.3)	.766	1.053(.748–1.483)
	**No-Lobular Special Type BC**	131 (7.6)	19 (4.4)	.589	.871(.529–1.436)
**Arrangement**	**Unilateral**	1674 (97.3)	409 (93.8)	**.004**	**2.265(1.306–3.927)**
	**Bilateral**	47 (2.7)	27 (6.2)		
**Location of BC**	**Left**	841 (48.9)	201 (46.1)	1	(Reference)
	**Bilateral**	47 (2.7)	27 (6.2)	**.004**	**2.265(1.306–3.927)**
	**Multicentric**	114 (6.6)	51 (11.7)	**.053**	**.874(762–1.001)**
**T Stage**	**T1**	648 (37.7)	66 (15.1)	1	(Reference)
	**T2**	889 (51.7)	237 (54.4)	**.003**	**1.737(1.214–2.486)**
	**T3**	131 (7.6)	45 (10.3)	.156	1.417(.875–2.295)
	**T4**	53 (3.1)	88 (20.2)	**.015**	**2.059(1.154–3.675)**
**N Stage**	**N0**	818 (47.5)	82 (18.8)	1	(Reference)
	**N1**	478 (27.8)	86 (19.7)	.226	1.304(.849–2.002)
	**N2**	293 (17)	154 (35.3)	.269	1.349(.793–2.296)
	**N3**	132 (7.7)	114 (26.1)	.232	1.396(.808–2.413)
**Stage**	**I**	422 (24.5)	23 (5.3)	1	(Reference)
	**II**	825 (47.9)	106 (24.3)	.433	1.265(.703–2.279)
	**III**	469 (27.3)	164 (37.6)	**.021**	**2.275(1.129–4.583)**
	**IV**	5 (0.3)	143 (32.8)	**< .001**	**32.105(15.256–67.563)**
**ER**	**Positive**	1393 (80.9)	319 (73.2)	**.086**	**1.688(.928–3.071)**
	**Negative**	328 (19.1)	117 (26.8)		
**PR**	**Positive**	1167 (67.8)	254 (58.3)	.651	1.071(.796–1.442)
	**Negative**	554 (32.2)	182 (41.7)		
**HER2**	**Positive**	383 (22.3)	125 (28.7)		**.536(.358-.802)**
				**.002**	

**Ki67**	**<15**	670 (39)	84 (19.3)	.622	1.076(.804–1.440)
	**≥15**	1049 (61)	352 (80.7)		
**Mitotic Index**	**1**	342 (19.9)	21 (4.8)	1	(Reference)
	**2**	913 (53.1)	160 (36.7)	**.009**	**1.951(1.180–3.224)**
	**3**	466 (27.1)	255 (58.5)	**< .001**	**2.753(1.607–4.715)**
**Tm Grade**	**Grade I**	261 (15.2)	28 (6.4)	1	(Reference)
	**Grade II**	845 (49.1)	180 (41.3)	.154	1.416(.878–2.283)
	**Grade III**	615 (35.7)	228 (52.3)	.384	1.257(.751–2.102)
**Skin Infiltration**	**Present**	82 (4.8)	86 (19.7)	.237	.753(.470–1.205)
	**N/A**	1639 (95.2)	350 (80.3)		
**EIC**	**N/A**	1459 (84.8)	340 (78)	.112	1.243(.950–1.627)
	**Present**	262 (15.2)	96 (22)		
**Subgroup**	**HER2 Enriched**	100 (5.8)	50 (11.5)		
	**Triple Negative**	199 (11.6)	54 (12.4)		
	**Luminal A**	547 (31.8)	65 (14.9)		
	**Luminal B**	875 (50.8)	267 (61.2)		
**Breast Surgery Type**	**BCS**	970 (56.6)	127 (34.7)	.356	1.131(.871–1.468)
	**MRM**	743 (43.4)	239 (65.3)		
**Axillary Surgery Type**	**SLND**	481 (28.1)	47 (12.8)	.267	.813(.563–1.172)
	**AK**	1232 (71.9)	319 (87.2)		
**Anti-Hormonal Treatment**	**Present**	1419 (82.5)	332 (76.1)	.437	.737(.342–1.590)
	**N/A**	302 (17.5)	104 (23.9)		
**Duration of TAM Use**	**N/A**	1059 (61.5)	190 (43.6)	1	(Reference)
		570 (33.1)	234 (53.7)	**.021**	**1.499(1.062–2.117)**
	**≤ 5 years**				
	**>5 years**	92 (5.3)	12 (2.8)	.642	.847(.421–1.704)
**Duration of AI Use**	**N/A**	669 (38.9)	196 (45)	1	(Reference)
	**≤ 5 years**	819 (47.6)	206 (47.2)	.249	.824(.593–1.145)
	**>5 years**	233 (13.5)	34 (7.8)	**.047**	**.607(.371-.993)**
**RT**	**Present**	1579 (91.7)	306 (70.2)	.524	.631(.153–2.607)
	**N/A**	142 (8.3)	130 (29.8)		
**RT Type**	**N/A**	134 (7.8)	127 (29.1)	1	(Reference)
	**Breast Only**	580 (33.7)	47 (10.8)	.222	.397(.090–1.747)
	**Locoregional**	1007 (58.5)	262 (60.1)	.174	.367(.086–1.558)
**Herceptin Eligibility**	**Eligible**	331 (19.2)	92 (21.1)	**.032**	**1.638(1.043–2.572)**
	**Ineligible**	1390 (80.8)	344 (78.9)		
**CT Received**	**N/A**	318 (18.5)	46 (10.6)	1	(Reference)
	**Neadjuvant**	184 (10.7)	82 (18.8)	.586	1.151(.694–1.911)
	**Adjuvant**	1219 (70.8)	308 (70.6)	.119	.708(.459–1.093)

ER: Estrogen Receptor, PR: Progesterone Receptor, HER2: Human Epidermal Growth Factor Receptor 2, EIC: Extensive TMX: Tamoxifen, AI: Aromatase Inhibitor, RT: Radiotherapy, CT: Chemotherapy, AC: Axillary Curettage SLND: Sentinel Lymph Node Dissection

Multivariate factors that were significant risk factors for OS were ILC histopathology (p = .045), T stage, N stage, stage, skin infiltration, positive surgical margins, high histological grade and mitotic index. MRM, chemotherapy (CT), radiotherapy (RT) and use of TAM and AI for more than 5 years were significant protective factors for OS (Tables 6 and 7).

**Table 6:** Univariate analysis results affecting OS.

		Survival Status [n (%)]		Univariate Cox Regression	
		Alive	Deceased	p	HR (95% CI)
**Age**	**<35**	87 (4.9)	25 (6.5)	1	(Reference)
	**36–50**	722 (40.8)	117 (30.2)	**.013**	**.577(.375-.889)**
	**>50**	961 (54.3)	245 (63.3)	.894	1.028(.681–1.552)
**Gender**	**Female**	1761 (99.5)	381 (98.4)	**.014**	**2.753(1.228–6.168)**
	**Male**	9 (0.5)	6 (1.6)		
**Menstruation State**	**Premenopausal**	718 (40.8)	118 (31)	**< .001**	**1.664(1.338–2.068)**
	**Postmenopausal**	1043 (59.2)	263 (69)		
**Family History**	**Present**	536 (30.3)	91 (23.5)	**.005**	**1.402(1.108–1.773)**
	**N/A**	1234 (69.7)	296 (76.5)		
**Histological Subtype Group- 1**	**No-Special Type**	1486	328	.379	.883(.669–1.165)
	**Special Type**	284	59		
**Histological Subtype Group- 2**	**No-Special Type**	1486	328 (84.7)	1	(Reference)
	**Invasive Lobular BC**	150	43 (11.1)	.502	1.115(.811–1.532)
	**No-lobular Special Type BC**	134 (7.6)	16 (4.1)	**.026**	**.566(.343-.935)**
**Arrangement**	**Unilateral**	1713 (96.8)	370 (95.6)	.690	1.104(.679–1.795)
	**Bilateral**	57 (3.2)	17 (4.4)		
**Breast**	**Left**	855 (48.3)	187 (48.3)	1	(Reference)
	**Right**	858 (48.5)	183 (47.3)	.695	.960(.783–1.177)
	**Bilateral**	57 (3.2)	17 (4.4)	.757	1.082(.658–1.777)
**Tumor Quadrant**	**Inner**	356 (20.1)	79 (20.4)	1	(Reference)
	**Outer**	1052 (59.4)	226 (58.4)	.962	.994(.769–1.284)
	**Areola**	234 (13.2)	45 (11.6)	.667	.923(.640–1.331)
	**Multicentric**	128 (7.2)	37 (9.6)	.074	1.428(.966–2.110)
**T Stage**	**T1**	654 (36.9)	60 (15.5)	1	(Reference)
	**T2**	920 (52)	206 (53.2)	**< .001**	**2.212(1.659–2.949)**
	**T3**	134 (7.6)	42 (10.9)	**< .001**	**2.636(1.777–3.912)**
	**T4**	62 (3.5)	79 (20.4)	**< .001**	**13.115(9.330–18.436)**
**N Stage**	**N0**	804 (45.4)	96 (24.8)	1	(Reference)
	**N1**	490 (27.7)	74 (19.1)	.338	1.160(.856–1.571)
	**N2**	319 (18)	128 (33.1)	**< .001**	**3.398(2.607–4.429)**
	**N3**	157 (8.9)	89 (23)	**< .001**	**4.318(3.234–5.764)**
**Stage**	**I**	420 (23.7)	25 (6.5)	1	(Reference)
	**II**	812 (45.9)	119 (30.7)	**< .001**	**2.113(1.373–3.252)**
	**III**	470 (26.6)	163 (42.1)	**< .001**	**4.783(3.139–7.286)**
	**IV**	68 (3.8)	80 (20.7)	**< .001**	**21.748(13.828–34.204)**
**Metastatic Site**	**N/A**	1621 (91.6)	125 (32.3)	1	(Reference)
	**Bone**	69 (3.9)	77 (19.9)	**< .001**	**9.199(6.922–12.226)**
	**Lung**	9 (0.5)	14 (3.6)	**< .001**	**10.972(6.311–19.075)**
	**Liver**	4 (0.2)	10 (2.6)	**< .001**	**17.278(9.058–32.959)**
	**Brain**	2 (0.1)	22 (5.7)	**< .001**	**26.432(16.733–41.753)**
	**Multiple**	65 (3.7)	139 (35.9)	**< .001**	**15.046(11.794–19.193)**
**Surgery**	**N/A**	30 (1.7)	48 (12.4)	**< .001**	**.091(.067-.125)**
	**Present**	1740 (98.3)	339 (87.6)		
**Breast Surgery Type**	**BCS**	998 (57.4)	99 (29.2)	**< .001**	**2.385(1.887–3.016)**
	**MRM**	742 (42.6)	240 (70.8)		
**Axillary Surgery**	**N/A**	30 (1.7)	48 (12.4)	**< .001**	**.091(.067-.125)**
	**Present**	1740 (98.3)	339 (87.6)		
**Axillary Surgery** **Type**	**SLND**	497 (28.6)	31 (9.1)	**< .001**	**2.298(1.586MNNMZ3.331)**
	**AC**	1243 (71.4)	308 (90.9)		
**Skin Infiltration**	**Present**	86 (4.9)	82 (21.2)	**< .001**	**.170(.132-.218)**
	**N/A**	1684 (95.1)	305 (78.8)		
**Surgical Margin**	**Negative**	1441 (81.4)	311 (80.4)	**.023**	**1.340(1.042–1.723)**
	**Positive**	329 (18.6)	76 (19.6)		
**Tm Grade**	**Grade I**	286 (16.2)	3 (0.8)	1	(Reference)
	**Grade II**	866 (48.9)	159 (41.1)	**< .001**	**15.313(4.886–47.986)**
	**Grade III**	618 (34.9)	225 (58.1)	**< .001**	**30.243(9.680–94.487)**
**Mitotic Index**	**1**	360 (20.3)	3 (0.8)	1	(Reference)
	**2**	918 (51.9)	155 (40.1)	**< .001**	**18.444(5.884–57.817)**
	**3**	492 (27.8)	229 (59.2)	**< .001**	**51.199(16.389–159.950)**
**ER**	**Positive**	1438 (81.2)	274 (70.8)	**< .001**	**1.675(1.345–2.085)**
	**Negative**	332 (18.8)	113 (29.2)		
**PR**	**Positive**	1194 (67.5)	227 (58.7)	**< .001**	**1.537(1.255–1.881)**
	**Negative**	576 (32.5)	160 (41.3)		
**HER2**	**Positive**	406 (22.9)	102 (26.4)	**.002**	**.697(.555-.876)**
	**Negative**	1364 (77.1)	285 (73.6)		
**Ki67**	**<15**	681 (38.5)	73 (18.9)	**< .001**	**2.493(1.932–3.217)**
	**≥15**	1087 (61.5)	314 (81.1)		
**EIC**	**N/A**	1502 (84.9)	297 (76.7)	**< .001**	**1.678(1.325–2.125)**
	**Present**	268 (15.1)	90 (23.3)		
**LVI**	**Present**	827 (46.7)	198 (51.2)	.078	.836(.685–1.020)
	**N/A**	943 (53.3)	189 (48.8)		
**PNI**	**Present**	314 (17.7)	86 (22.2)	.313	.884(.695–1.123)
	**N/A**	1456 (82.3)	301 (77.8)		
**Subgroup Luminal**	**Luminal A-B**	1468 (82.9)	286 (73.9)	1	(Reference)
	**Triple Negative**	198 (11.2)	55 (14.2)	**.034**	**1.366(1.023–1.823)**
	**HER2 Enriched**	104 (5.9)	46 (11.9)	**< .001**	**2.415(1.767–3.301)**
**Subgroup**	**HER2 Enriched**	104 (5.9)	46 (11.9)	1	(Reference)
	**Triple Negative**	198 (11.2)	55 (14.2)	**.004**	**.565(.381-.836)**
	**Luminal A**	559 (31.6)	53 (13.7)	**< .001**	**.209(.141-.310)**
	**Luminal B**	909 (51.4)	233 (60.2)	**< .001**	**.532(.387-.731)**
**Anti-Hormonal Treatment**	**Present**	1466 (82.8)	285 (73.6)	**< .001**	**1.723(1.374–2.160)**
	**N/A**	304 (17.2)	102 (26.4)		
**Duration of TAM Use**	**N/A**	1034 (58.4)	215 (55.6)	1	(Reference)
	**≤ 5 years**	640 (36.2)	164 (42.4)	.938	1.008(.822–1.237)
	**>5 years**	96 (5.4)	8 (2.1)	**.001**	**.294(.145-.595)**
**Duration of AI Use**	**N/A**	691 (39)	174 (45)	1	(Reference)
	**≤ 5 years**	832 (47)	193 (49.9)	.116	.848(.691–1.041)
	**>5 years**	247 (14)	20 (5.2)	**< .001**	**.237(.149-.377)**
**Herceptin Eligibility**	**Eligible**	361 (20.4)	62 (16)	.665	.941(.715–1.239)
	**Ineligible**	1409 (79.6)	325 (84)		
**RT**	**Present**	1591 (89.9)	294 (76)	**< .001**	**2.380(1.884–3.006)**
	**N/A**	179 (10.1)	93 (24)		
**RT Type**	**N/A**	171 (9.7)	90 (23.3)	1	(Reference)
	**Breast Only**	574 (32.4)	53 (13.7)	**< .001**	**.236(.168-.331)**
	**Locoregional**	1025 (57.9)	244 (63)	**< .001**	**.506(.397-.645)**
**CT**	**N/A**	316 (17.9)	48 (12.4)	1	(Reference)
	**Neadjuvant**	206 (11.6)	60 (15.5)	**.002**	**1.839(1.258–2.689)**
	**Adjuvant**	1248 (70.5)	279 (72.1)	.864	1.027(.756–1.396)
**CT Type**	**N/A**	333 (18.8)	55 (14.2)	1	(Reference)
	**AC+TXT**	888 (50.2)	128 (33.1)	.503	.898(.654–1.232)
	**FAC-FEC-TAC**	312 (17.6)	130 (33.6)	.378	1.154(.839–1.587)
	**FAC-FEC+TXT**	185 (10.5)	48 (12.4)	.815	.955(.647–1.408)
	**RIBO+PABLO**	5 (0.3)	1 (0.3)	.268	3.061(.423–22.183)
	**PERJETA**	20 (1.1)	2 (0.5)	.905	1.090(.265–4.476)
	**CMF**	27 (1.5)	23 (5.9)	**< .001**	**2.627(1.613–4.279)**

ER: Estrogen Receptor, PR: Progesterone Receptor, HER2: Human Epidermal Growth Factor Receptor 2, EIC: Extensive Intraductal Carcinoma, LVI: Lymphovascular Invasion, PNI: Perineural Invasion, TMX: Tamoxifen, AI: Aromatase Inhibitor, RT: Radiotherapy, CT: Chemotherapy, AC: Axillary Curettage, SLND: Sentinel Lymph Node Dissection, AC: Adriamycin, Cyclophosphamide, TXT: Taxotere, FAC: Cyclophosphamid, Adriamycin, 5-Fulourouracil, FEC: 5-Fulouracil, Epirubicine, Cyclophosphamide, TAC: Taxotere, Adriamycin, Cyclophosphamid, RIBO+PABLO:Ribociclib+Palbociclib, CMF: Cyclophosphamide, Methotrexate, Fluorouracil

**Table 7:** Multivariate analysis results affecting OS.

		Survival Status		Multivariate Cox Regression	
		Alive	Deceased	p	HR (95% CI)
**Age**	**<35**	87 (4.9)	25 (6.5)	1	(Reference)
	**36–50**	722 (40.8)	117 (30.2)	.555	1.185(.675–2.079)
	**>50**	961 (54.3)	245 (63.3)	.168	1.578(.825–3.019)
**Menstruation Status**	**Premenopausal**	718 (40.8)	118 (31)	.981	1.005(.675–1.495)
	**Postmenopausal**	1043 (59.2)	263 (69)		
**Family History**	**Present**	536 (30.3)	91 (23.5)	.191	1.205(.911–1.593)
	**N/A**	1234 (69.7)	296 (76.5)		
**Histological Subtype Group—2**	**No-Special Type**	1486	328 (84.7)	1	(Reference)
	**Invasive Special Lobular BC**	150	43 (11.2)	**.045**	**1.457(1.009–2.104)**
	**No-Lobular Special Type BC**	134 (7.6)	16 (4.1)	.579	.861(.507–1.462)
**T Stage**	**T1**	654 (36.9)	60 (15.5)	1	(Reference)
	**T2**	920 (52)	206 (53.2)	.191	1.301(.877–1.929)
	**T3**	134 (7.6)	42 (10.9)	**.021**	**1.791(1.094–2.932)**
	**T4**	62 (3.5)	79 (20.4)	**.005**	**2.486(1.315–4.700)**
**N Stage**	**N0**	804 (45.4)	96 (24.8)	1	(Reference)
	**N1**	490 (27.7)	74 (19.1)	.790	1.056(.707–1.578)
	**N2**	319 (18)	128 (33.1)	**.002**	**2.358(1.384–4.018)**
	**N3**	157 (8.9)	89 (23)	**.007**	**2.184(1.243–3.836)**
**Stage**	**I**	420 (23.7)	25 (6.5)	1	(Reference)
	**II**	812 (45.9)	119 (30.7)	**.058**	**1.802(.980–3.314)**
	**III**	470 (26.6)	163 (42.1)	.825	1.090(.507–2.343)
	**IV**	68 (3.8)	80 (20.7)	.112	1.987(.853–4.631)
**Skin Infiltration**	**Present**	86 (4.9)	82 (21.2)	**.034**	**.598(.372-.961)**
	**N/A**	1684 (95.1)	305 (78.8)		
**Surgical Margin**	**Negative**	1441 (81.4)	311 (80.4)	**< .001**	**1.666(1.275–2.176)**
	**Positive**	329 (18.6)	76 (19.6)		
**Duration of TAM Use**	**N/A**	1034 (58.4)	215 (55.6)	1	(Reference)
	**≤ 5 years**	640 (36.2)	164 (42.4)	**< .001**	**.430(.295-.625)**
	**>5 years**	96 (5.4)	8 (2.1)	**< .001**	**.177(.074-.423)**
**Duration of AI Use**	**N/A**	691 (39)	174 (45)	1	(Reference)
	**≤ 5 years**	832 (47)	193 (49.9)	**.015**	**.609(.407-.909)**
	**>5 years**	247 (14)	20 (5.2)	**< .001**	**.143(.077-.266)**
**ER**	**Positive**	1438 (81.2)	274 (70.8)	.395	1.307(.706–2.419)
	**Negative**	332 (18.8)	113 (29.2)		
**PR**	**Positive**	1194 (67.5)	227 (58.7)	.584	.910(.650–1.275)
	**Negative**	576 (32.5)	160 (41.3)		
**HER2**	**Positive**	406 (22.9)	102 (26.4)	.197	.790(.552–1.130)
	**Negative**	1364 (77.1)	285 (73.6)		
**Ki67**	**<15**	681 (38.5)	73 (18.9)	.502	.836(.496–1.410)
	**≥15**	1087 (61.5)	314 (81.1)		
**Mitotic Index**	**1**	360 (20.3)	3 (0.8)	1	(Reference)
	**2**	918 (51.9)	155 (40.1)	**.025**	**2.616(1.131–6.053)**
	**3**	492 (27.8)	229 (59.2)	**.005**	**3.530(1.475–8.449)**
**Tm Grade**	**Grade I**	286 (16.2)	3 (0.8)	1	(Reference)
	**Grade II**	866 (48.9)	159 (41.1)	.091	2.080(.889–4.868)
	**Grade III**	618 (34.9)	225 (58.1)	**.025**	**2.748(1.139–6.631)**
**Chemotherapy**	**N/A**	316 (17.9)	48 (12.4)	1	(Reference)
	**Neoadjuvant**	206 (11.6)	60 (15.5)	**< .001**	**.141(.053-.373)**
	**Adjuvant**	1248 (70.5)	279 (72.1)	**< .001**	**.161(.066-.392)**
**EIC**	**N/A**	1502 (84.9)	297 (76.7)	.281	1.180(.873–1.595)
	**Present**	268 (15.1)	90 (23.3)		
**Breast Surgery Type**	**BCS**	998 (57.4)	99 (29.2)	**.008**	**1.546(1.122–2.130)**
	**MRM**	742 (42.6)	240 (70.8)		
**Axillary Surgery**	**SLND**	497 (28.6)	31 (9.1)	.099	1.443(.933–2.234)
**Type**	**AK**	1243 (71.4)	308 (90.9)		
**RT**	**Present**	1591 (89.9)	294 (76)	.122	2.447(.788–7.600)
	**N/A**	179 (10.1)	93 (24)		
**RT Type**	**N/A**	171 (9.7)	90 (23.3)	1	(Reference)
	**Breast Only**	574 (32.4)	53 (13.7)	**.026**	**3.729(1.167–11.913)**
	**Locoregional**	1025 (57.9)	244 (63)	.431	1.609(.492–5.262)
**Site of Metastasis**	**N/A**	1621 (91.6)	125 (32.3)	1	(Reference)
	**Bone**	69 (3.9)	77 (19.9)	**< .001**	**9.010(6.481–12.524)**
	**Lung**	9 (0.5)	14 (3.6)	**< .001**	**10.887(6.034–19.644)**
	**Liver**	4 (0.2)	10 (2.6)	**< .001**	**14.829(6.774–32.464)**
	**Brain**	2 (0.1)	22 (5.7)	**< .001**	**26.076(15.484–43.914)**
	**Multiple**	65 (3.7)	139 (35.9)	**< .001**	**11.479(5.457–17.249)**

ER: Estrogen Receptor, PR: Progesterone Receptor, HER2: Human Epidermal Growth Factor Receptor 2, EIC: Extensive Intraductal Carcinoma, LVI: Lymphovascular Invasion, PNI: Perineural Invasion, TMX: Tamoxifen, AI: Aromatase Inhibitor, RT: Radiotherapy, CT: Chemotherapy, AC: Axillary Curettage, SLND: Sentinel Lymph Node Dissection

## Discussion

The classification of special types of BC recommended by the World Health Organization is beginning to take a wider place in literature because of their distinct biological behavior and clinical characteristics compared to No-Special Types of BC [15]. Additionally, subtypes in the special BC group may behave very differently from each other. ILC is considered notable for its distinctive biological behavior and unusual organ metastases and is included in Special Type BC because of studies showing it has a better prognosis than IDC [6, 20–24]. However, studies with longer periods of follow-up show that ILC has worse prognosis than IDC [6, 14, 25]. Survival data, which differ from each other and change over the years, call into question the status of ILC in the Special Type BC group. A separate classification may be necessary for subtypes in this group.

When the patients in our study were divided into two subgroups (No-Special Type BC, Special Type BC), no significant difference was observed regarding DFS and OS. When the data was later reanalyzed by removing ILC from the Special Type BC and treating it as a third, distinct subgroup, Invasive Lobular Special Type BC was found to have the lowest duration of DFS and OS. There was a significant difference between the duration of DFS and OS of No-Special Type BC and No-Lobular Special Type BC. The duration of DFS and OS showed significant difference in Invasive Lobular Special Type BC and No-Lobular Special Type BC as well. Although No-Special Type BC and Invasive Lobular Special Type BC did not have a statistically significant difference regarding DFS and OS, the difference was still remarkable. No-Special Type BC and Invasive Lobular Special Type BC have similar durations of DFS in the first 6 years. After 6 years however, the survival curve for Invasive Lobular Special Type BC was lower than of No-Special Type BC and the difference becomes more pronounced after the 17^th^ year. OS for Invasive Lobular Special Type BC showed a lower course than No-Special Type BC after 14 years. Although the difference between the duration of OS in Invasive Lobular Special Type BC and No-Special Type BC were not statistically significant, Invasive Lobular Special Type BC significantly increased the risk of death by 1.457(1.009–2.104) times, (*p =* .*045*) in the Cox regression risk analysis.

Invasive Lobular Special Type-BC has a lower incidence, which may have resulted in limited knowledge of the clinical and biological features of the histopathological subtypes within the group. However, recent studies with longer follow-up periods have reported a lower survival rate, especially for Invasive Lobular Special Type-BC, contrary to current information [14, 26]. The increase of incidence and low survival rates may force the clinicians to reconsider treatment options and the frequency of follow-ups.

A study by Toikkanen et al. [7] showed ILC had better prognosis than IDC despite 30 years of follow-up, which precludes interpretation by the length of follow-up alone. As a result, when ILC is analyzed yearly, an increase in its incidence is seen. Earlier studies [1, 2, 4, 7] show ILC to have lower grade, mitotic activity, T stage, N stage, stage, and ER positivity with a higher rate of bilateral arrangement, while more recent studies [14, 26] report higher histologic grade and mitotic activity, and diagnosis at more advanced stages. Our study shows that ILC is associated with larger tumor size, older age, more advanced T and N stage, lower grade, higher ER and PR positivity, and lower HER2 expression, while the rate of bilateral arrangement was not higher. This may suggest that the biological behavior of ILC has become more aggressive over the years. It should be noted that the lack of difference between IDC and ILC in terms of surgical margin positivity, breast surgery and axillary surgery type in our series is evidence that it does not show a worse prognosis due to residual disease or incomplete treatment. In the multivariate analysis for DFS, neither breast nor axillary surgery type increased the risk of events. In OS, the risk increased 1.5 times at the statistical significance level (*p = 0*.*008*) in patients who underwent MRM. In the axillary surgery type, the risk increased by 1.4 in patients who underwent AC which was close to statistical significance (*p = 0*.*099*). Since more radical surgical interventions are already preferred in patients with higher risk, incomplete surgery or less aggressive surgical methods should not be labeled as the cause of shorter survival and worse prognosis in ILC when interpreting the results in our series with a peace of mind.

Recent studies suggest whether IDC is the most common histopathological subgroup with the worst prognosis should be re-evaluated. Having the lowest course on the survival curve, patients with ILC may require a more careful approach. Our study suggests that reviewing the current treatment guidelines may be required. Increased incidence of ILC may be linked to improved diagnostic approach. Whether ILC has a better or similar prognosis and higher rates of bilateral arrangement in comparison to IDC deserves restatement.

Studies in which the pathological complete response to neoadjuvant chemotherapy is rare and positive surgical margin rate is higher in ILC [13, 22, 26]. This uncertainty may have made mastectomy a more common treatment option for ILC than IDC [6, 7, 14]. In another retrospective series, with only larger tumor size as a poor prognostic factor, ILC had lower survival compared to IDC [6]. Pestalozzi et al. [14], on the other hand, associated the difference in survival with stage and reported that the early-stage prognosis of ILC is better than IDC, but the late-stage prognosis of ILC is worse [25]. Since ILC presents itself with diffuse or spreading lesions rather than a mass, it has been stated that breast MRI is more helpful than mammography in the diagnosis of such lesions being satisfied with mammography alone may cause delays in the diagnosis of ILC [26, 27]. Failure to encode the E-Cadherin protein encoded by the CHD1 gene due to somatic mutation is a pathognomonic condition for ILC compared to IDC. The E-Cadherin protein not only plays a role in the adhesion of cells to each other to form tissues and in the transmission of chemical signals within the cell, cell maturation and controlling cell movement, it also functions as a tumor suppressor protein [28–30]. Deletion of E-Cadherin and PI3K pathways may be the cause of infiltrative growth pattern and frequent surgical margin positivity [31, 32].

Precisely for this reason, the results of the ROSALINE (NCT04551495) phase II study with entrectinib, a tyrosine kinase inhibitor targeting TRK, ROS1 and ALK tyrosine kinases, with promising in vivo results are eagerly awaited. It is aimed to measure the antitumor efficacy of entrectinib and endocrine therapy as neoadjuvant in ER-positive, HER2-negative ILC BC patients [33]. For patients with metastatic ILC, mutations in CDH1, NF1, PIK3CA, and TBX3, measured as tumor mutational burden (TMB), are higher than for patients with metastatic IDC. The effect of atezolizumab, a carboplatin and immune checkpoint inhibitors (ICIs), will be measured in the GELATO study, which is planned for metastatic lobular BC assessing efficacy with mutational burden (TMB) [34].

The limitation of our study is that, apart from a retrospective series and comparison of clinical features and treatment outcomes for ILC, no additional molecular comparison could be made. However, pre-clinical and clinical molecular studies have been and continue to be conducted in order to explain the clinical process difference in ILC. The fact that the reason for the different clinical course and biology of the ILC subtype of BC has begun to be revealed raises our hopes that the treatment options will also differ in the coming years and that the risk of developing late recurrence and metastasis will be reduced or eliminated with more effective treatments.

## Conclusion

The increase in the incidence of the ILC histopathological subgroup of BC in the recent years has not only made it possible to understand that it has a different course compared to IDC but has also caused systemic and local treatment decisions to be different over time. In our study and other studies with long durations of follow-up, it is seen that ILC has the lowest DFS and OS among all histological subgroups. Therefore, the classification of ILC into Special Type BC should be reassessed and current treatment guidelines may need to be revised, particularly as ILC-specific study results begin to emerge.
